# The Disruption of Geniculocalcarine Tract in Occipital Neoplasm: A Diffusion Tensor Imaging Study

**DOI:** 10.1155/2016/8213076

**Published:** 2016-08-16

**Authors:** Yan Zhang, Sihai Wan, Ge Wen, Xuelin Zhang

**Affiliations:** ^1^Zhongshan Ophthalmic Center, State Key Laboratory of Ophthalmology, Sun Yat-sen University, Guangzhou, Guangdong 510060, China; ^2^Department of Radiology, Lu Shan Sanatorium, Nanjing Military Region, Jiujiang, Jiangxi 332000, China; ^3^Department of Radiology, Nanfang Hospital, Southern Medical University, Guangzhou, Guangdong 510515, China

## Abstract

*Aim*. Investigate the disruption of geniculocalcarine tract (GCT) in different occipital neoplasm by diffusion tensor imaging (DTI).* Methods*. Thirty-two subjects (44.1 ± 3.6 years) who had single occipital neoplasm (9 gliomas, 6 meningiomas, and 17 metastatic tumors) with ipsilateral GCT involved and thirty healthy subjects (39.2 ± 3.3 years) underwent conventional sequences scanning and diffusion tensor imaging by a 1.5T MR scanner. The diffusion-sensitive gradient direction is 13. Compare the fractional anisotropy (FA) and mean diffusivity (MD) values of healthy GCT with the corresponding values of GCT in peritumoral edema area. Perform diffusion tensor tractography (DTT) on GCT by the line propagation technique in all subjects.* Results*. The FA values of GCT in peritumoral edema area decreased (*P* = 0.001) while the MD values increased (*P* = 0.002) when compared with healthy subjects. There was no difference in the FA values across tumor types (*P* = 0.114) while the MD values of GCT in the metastatic tumor group were higher than the other groups (*P* = 0.001). GCTs were infiltrated in all the 9 gliomas cases, with displacement in 2 cases and disruption in 7 cases. GCTs were displaced in 6 meningiomas cases. GCTs were displaced in all the 7 metastatic cases, with disruption in 7 cases.* Conclusions*. DTI represents valid markers for evaluating GCT's disruption in occipital neoplasm. The disruption of GCT varies according to the properties of neoplasm.

## 1. Introduction

The eloquent white matter tracts can be delineated by diffusion tensor imaging (DTI) in patients with intracranial neoplasm. On the one hand, the exact relative position between the neoplasm and white matter tracts can be investigated and prompted for dysfunction [1–4]. For instance, motor disability with neoplasm related to the corticospinal tract has been investigated. DTT could visualize the exact location of tumors relevant to eloquent tracts and was found to be beneficial in the neurosurgical planning and postoperative assessment [5, 6]. On the other hand, diffusion indices may prompt neoplasm's histopathology type, tumor fraction, and axonal disruption of fiber tracts since their changes provide information for the underlying microanatomic changes or pathological changes [7–11].

Geniculocalcarine tract (GCT) is a fiber tract that originates from lateral geniculate body (LGB), passes through the sublenticular internal capsule and lenticular nucleus, along the lateral sagittal plane besides the occipital horn, and shapes like a convex lamina, and finally terminates in the calcarine fissure of occipital lobe. It conducts nervous impulse from LGB to the primary visual cortex in occipital lobe. Investigating the disruption of GCT in different occipital neoplasm may assist in identifying conditions occult to structural imaging and provide relational information that is critical to clinical decision making [12–15]. For instance, Salmela et al. used optic nerve tractography to aid in surgical planning for pediatric suprasellar tumors [15]. However, GCT involved in occipital neoplasm has not been delineated well in general.

In this research, we investigated GCTs in different occipital neoplasm by DTI and assumed that diffusion indices were valid markers in evaluating GCTs' disruption. We compared fractional anisotropy (FA) and mean diffusivity (MD) values of healthy GCTs with corresponding values of GCTs in peritumoral edema area and performed DTT on GCTs in all subjects.

## 2. Materials and Methods

### 2.1. Standard Protocol Approvals and Patient Consent

The research protocol was approved by the ethics committees for clinical research. All of the procedures involving the participants were conducted following the Declaration of Helsinki and institutional guidelines in compliance with the stated regulations. Written informed consent was obtained from all of the participants.

### 2.2. Subjects

The study group consisted of 32 subjects (17 males and 15 females; age: 44.1 ± 3.6 years, range: 35–61) who had single occipital neoplasm with ipsilateral GCT involved. These cases included 9 gliomas (World Health Organization grade II, 2 cases; grades III and IV, 7 cases), 6 meningiomas, and 17 metastatic tumors (lung cancer, 14 cases; breast cancer, 2 cases; gastric cancer, 1 case). All cases were certified by pathological examinations of surgical specimens. 14 subjects had homonymous hemianopia in the half visual field contralateral to the neoplasm.

For the control group, 30 healthy volunteers (15 males and 15 females; age: 39.2 ± 3.3 years, range: 20–63) were recruited from the outpatients.

Inclusion criteria consisted of (1) being right handed, (2) in the study group, each subject having a single occipital neoplasm with ipsilateral GCT involved in conventional MR scanning, (3) in the control group, no occupied lesion or abnormal findings in conventional MR scanning, (4) no history of neurological diseases including cerebrovascular disease, neurodegenerative disease, and trauma in both groups, and (5) no drug, alcohol, or addictive substance abuse.

### 2.3. Data Acquisition

MRIs were performed using a 1.5-Tesla scanner (Signa Twin, GE, USA) with an 8-channel head-phased array coil. The baseline scan was in the axial plane. Head movement was limited by vacuum fixation cushions.

All the subjects underwent conventional sequences scanning, including T_1_-fluid attenuated inversion recovery (FLAIR), T_2_WI, and T_2_-FLAIR. Consecutive slices were acquired in all sequences. DTI was performed in a spin echo-echo planar imaging (SE-EPI) diffusion tensor sequence in the axial plane right after the conventional sequences scanning (*b* = 0/1000 s/mm^2^; diffusion-sensitive gradient direction = 13; voxel size = 0.9 mm × 0.9 mm × 0.9 mm). The acquisition parameters of each sequence were listed in Table 1.

### 2.4. Data Analysis

The neoplasm of each subject was defined in the occipital horn planes of T_1_WI and T_2_WI images.

**Figure 1:**
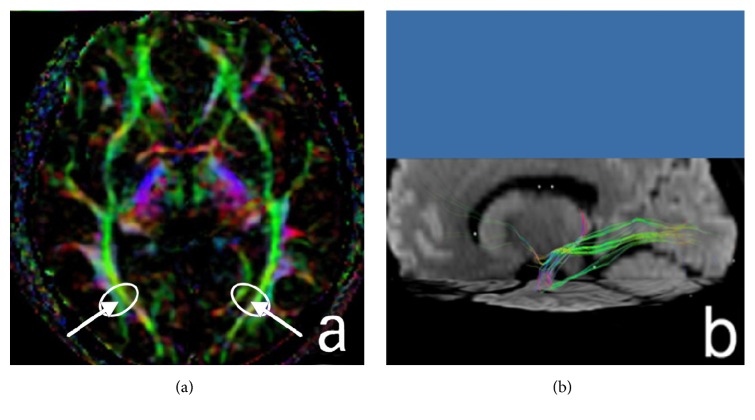
The GCT fibers ((a), white arrows) appear completely as green fiber tracts besides the paracele in healthy volunteers. It is a fiber tract from LGB and terminates in the calcarine fissure of occipital lobe (b).

**Figure 2:**
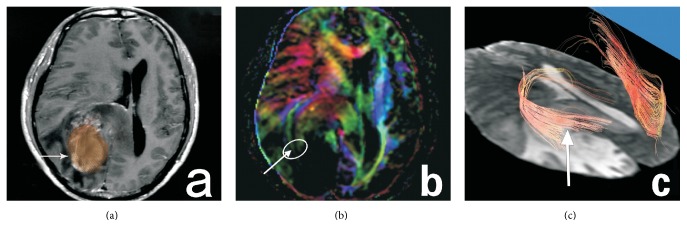
The contrast T_1_WI image of right occipital lobe gliomas in a 55-year-old man ((a), arrow). The FA value of the right GCT decreases significantly and it can not be identified in the DEC map ((b), arrow). DTT map shows disruption and infiltration of the right GCT fibers ((c), arrow).

**Figure 3:**
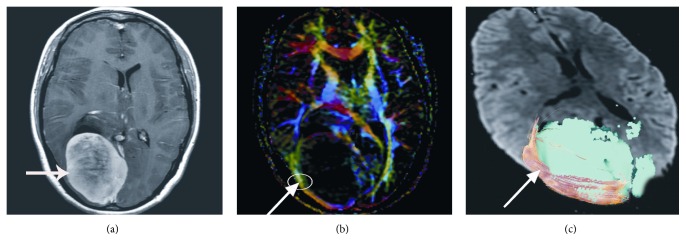
The contrast T_1_WI image of right occipital lobe meningioma in a 42-year-old woman ((a), arrow). The FA value of right GCT decreases mildly ((b), arrow). DTT map shows the right GCT fibers are displaced ((c), arrow).

**Figure 4:**
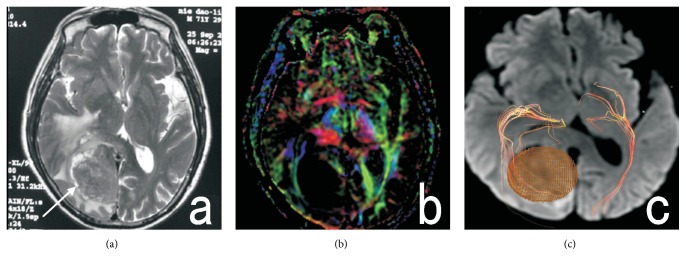
The T_2_WI image of right occipital lobe metastases in a 71-year-old man ((a), arrow). The FA value of right GCT decreases ((b), arrow). DTT map shows the right GCT fibers ((c), arrow) are disrupted by the metastasis (brown mass).

DTI datasets were processed using Volume One 1.72 (GE Healthcare, USA) and Diffusion Tensor Visualizer 1.72 software (Tokyo University, Tokyo, Japan). In the control group, region of interest (ROI) of GCT was drawn on the axial directionally encoded color (DEC) image in the occipital horn plane (Figure 1(a)). To investigate the diffusion indices of GCT in peritumoral edema area, select the most edematous area adjacent to neoplasm as the ROI in the study group (Figures 2(b), 3(b), and 4(b), circles). Obtain the FA and MD values of each ROI. Repeat the measurement by the same reader in three continuous slices, three ROIs each slice. Obtain the mean value of all the measurements as the final result of FA and MD values of each ROI.

Perform DTT on GCT by the line propagation techniques. The lateral geniculate body (LGB), which is located at the posterior lateral of thalamus, was taken as the seed point and the occipital lobe as the termination in the cross plane of DEC image. The termination of tractography was as follows: FA < 0.15, step < 160, and So < 120. Repeat the measurement by two readers with similar experience in DTI [16].

Witwer et al. [17] categorized the disruption of white matter (WM) tracts as edematous, displaced, and infiltrated in the following rules: (1) displaced: the WM tracts had abnormal pattern and location but normal FA values; (2) disrupted: the WM tracts disappeared in images with significant decrease in FA values; (3) infiltrated: the FA values decreased when compared with normal WM tracts. The WM tracts had abnormal pattern and location but they still can be identified by tractography.

### 2.5. Statistical Analysis

Two-sample *t*-test was used to compare the FA and MD values of GCT between the study group and the control group. One-way ANOVA was used to compare the FA and MD values of GCT in peritumoral edema area across tumor types. LSD *t*-test was used to compare the FA and MD values of GCT between each two types of neoplasm. Kappa-test was used to test the consistency of different readers in categorizing GCT's deformation. *P* < 0.05 was used to determine statistical significance. All analyses were performed by the Statistical Package for the Social Sciences software, Version 13.0 (SPSS, Chicago, Illinois, USA).

## 3. Results

The FA values of GCT in peritumoral edema area decreased (*P* = 0.001) while the MD values increased when compared with the control group (*P* = 0.002). The MD values of peritumoral edema area in the metastatic tumor group were higher than the other two groups (*P* = 0.001) while the FA values had no difference (*P* = 0.114) (Table 2).

The GCTs in healthy subjects were green fiber tracts besides the paracele triangular on DEC images (Figure 1).

GCT deformation differentiates in various types of neoplasm, which presents as round lesions in T_1_WI and T_2_WI images (Figures 2(a), 3(a), and 4(a), arrows). The two readers had consistency in categorizing GCTs deformation (*K* = 0.784). The GCTs in gliomas group were all infiltrated (9 cases). The GCTs in grade II gliomas were displaced (2 in 9 cases). But the GCTs in grades III and IV gliomas were disrupted (7 in 9 cases) (Figure 2(c)). The GCTs in meningiomas were compressed and shifted without disruption (Figure 3(c)). DTT images showed that GCT in metastases all had displacement (17 cases), and some companied with disruption (7 in 17 cases) (Figure 4(c)).

## 4. Discussion 

The FA values of GCT in peritumoral edema area decreased while the MD values increased when compared with the control group. FA value stands for the anisotropy of water molecules in each voxel. Decreased FA value may be due to the disorganization of axons and disruption of myelin sheath and the increase in extracellular water content [18]. In peritumoral edema area, the ordered axonal arrangements and the integrity of myelin sheath are damaged. The diffusion resistance decreases with the disintegration of these restrictive barriers. The water molecules lose their directivity parallel to the fiber tract and present in a chaotic state. The extracellular space and the water content of tissues increase in peritumoral edema area, which induce MD value to increase [19].

The FA values of GCT in peritumoral edema area had no difference across tumor types in our study (Table 2). Provenzale et al. made the same conclusion from their investigation. They found that the difference in FA decrease in peritumoral hyperintense regions was not significant between glioma and meningiomas [20]. Lu et al. also found that the peritumoral FA value did not differ significantly between high grade gliomas and metastatic tumors [21]. However, peritumoral fiber tracts alterations in gliomas were more complex as described so far since there were competing findings [20–22]. Investigating and categorizing peritumoral fiber tract alterations with FA values may not be credible enough to make a conclusion. Not only tumoral infiltration but also increase of water content in mesenchyme caused by the damage of blood brain barrier and blood-vessel osmotic increase can be found in the peritumoral hyperintensive regions of high grade gliomas. Using multimodel neuroimaging tools may provide more complementary information and understanding of it [22].

The MD values of GCT in peritumoral edema area in metastatic tumor group were higher than the other two groups (Table 2). This was in accordance with Lu et al.'s study that the peritumoral MD of metastatic lesions measured significantly greater than that of gliomas [21]. MD values increase with the water content of tissue and suggest vasogenic edema. The extracellular space also increases in the presence of elevated MD values. Some researchers considered that increased MD values suggest the infiltration of malignant cell, which destructed the ultrastructure in extracellular matrix and caused the movements of water molecules to be less restricted [23]. But most of researchers thought that a reliable differentiation between infiltration and vasogenic edema is not yet possible on the basis of DTI parameters [24, 25]. For instance, Tropine et al. determined that the FA and MD can not differentiate between accompanying edema and tumor cell infiltration of WM beyond the tumor edge in gliomas [26]. Kinoshita et al. also concluded that diffusion tensor-based tumor infiltration index cannot discriminate vasogenic edema from tumor-infiltrated edema from investigation with meningioma and glioma [27].

In our study, the disruption of GCT varied in different neoplasm. The pattern and location of displaced GCTs change but the fiber integrity keeps normal, which means the axons are not destructed. Displaced GCTs are mostly seen in meningioma. As the most frequent intracalvarium benign tumor is located outside the brain, meningioma shifts and compresses the brain but does not destroy WM tracts [28]. The function of GCT in subject with meningioma may recover when the lesion has been removed. Many metastatic tumors also cause the displacement of GCT when they are far away from it. Yet displacement always companies with disruption when the GCTs are in close relationship with metastatic tumors [29].

The FA values of disrupted GCT decreased significantly. This suggests a reduction in axonal density and arrangement of the GCT so its prognosis is pessimistic. The movement of water molecule along the fiber of GCT is constrained by the axon sheath [26, 29, 30]. The destruction of axons will cause water molecules to lose their directivity parallel to the fiber tract and present in a chaotic state, which leads to significant decrease of FA. WM tract disruption always develops in high grade (grades III and IV) gliomas and metastatic tumors. GCTs in high grade gliomas and metastatic tumors were disrupted with adjacent white matter infiltrated in our study.

Infiltration is a deformation between displacement and disruption. The infiltrated GCT has abnormal pattern and location with decreased FA values but still can be identified in FA images. This suggests that the axonal destructions are less serious than the disrupted ones. Infiltrated GCTs all developed in gliomas in our study. This may be because gliomas originate from myelin sheath gliocytes without specific boundary from normal nervous tissue. GCT is gradually destructed by malignant tumor cells in high grade gliomas.

Though the FA and MD in peritumoral edema area can not supply certain information for the involved tracts, they supply a direction to investigate tracts' alterations and potentially predictions of patients' prognosis [31, 32]. As a noninvasive technique to evaluate the WM integrity and fiber connectivity in vivo, DTI can assist neurosurgeons in identifying conditions occult to structural imaging and provide relational information that is critical to neurosurgery decision making [33]. Even if the classification of WM tracts' disruption in neoplasm lacks unified standard and seems to be oversimplified, DTT still acts as the most visual method for presenting function damage and estimating possible outcome from stereoscopic images of target WM tracts so far.

## 5. Conclusion

In this study, we investigated the disruption of GCT in different occipital neoplasm by DTI. The disruption of GCT varies according to the properties of neoplasm and can be categorized as disrupted, displaced, and infiltrated. DTI indices represent valid markers for GCT disruption in occipital lobe neoplasm.

## Figures and Tables

**Table 1:** The parameters of each acquisition sequence in MRI scanning.

Acquisition sequences	TR/TE (ms)	Matrix size	FOV (cm)	NEX	Slice thickness/interslice separation (mm)	Acquisition time (min:sec)
T_1_-FLAIR	2500/11.9	320 × 256	24 × 18	2	5/1.5	3:28
T_2_WI	4900/99.3	320 × 224	24 × 18	2	5/1.5	1:42
T_2_-FLAIR	8500/128	320 × 192	24 × 24	1	5/1.5	2:24
DTI SE-EPI	6000/60.1	128 × 128	24 × 24	2	3/0	6:52

**Table 2:** FA and MD of GCT in the study group and the control group (×10^−3^ mm^2^/s).

Control group		Study group								*P*
		(1) Gliomas	(2) Meningiomas	(3) Metastases	Total	*P*(1, 2, 3)	*P*(1-2)	*P*(1-3)	*P*(2-3)	
N	30	9	6	17	32					
FA	0.505 ± 0.028	0.205 ± 0.061	0.197 ± 0.028	0.171 ± 0.285	0.192 ± 0.125	0.114	0.978	0.306	0.245	0.001
MD	0.735 ± 0.047	1.379 ± 0.186	1.335 ± 0.202	1.695 ± 0.270	1.467 ± 0.218	0.001	0.964	0.003	0.015	0.002

*P* is the value of comparison between the study group and the control group.

*P*(1, 2, 3) is the value of comparison across tumor types.

## Abbreviations

DTI: Diffusion tensor imaging
DTT: Diffusion tensor tractography
GCT: Geniculocalcarine tract
FA: Fractional anisotropy
MD: Mean diffusivity
FLAIR: Fluid attenuated inversion recovery
SE-EPI: Spin echo-echo planar imaging
DEC: Directionally encoded color
ROI: Regions of interest
LGB: Lateral geniculate body
WM: White matter.
